# A Review of Guidelines/Guidance from Various Countries Around the World for the Prevention and Management of Travellers’ Diarrhoea: A Pharmacist’s Perspective

**DOI:** 10.3390/pharmacy7030107

**Published:** 2019-08-04

**Authors:** Geeta Hitch

**Affiliations:** Department of Life Sciences (Pharmacy), University of Sussex, Falmer, Brighton BN1 9RH, UK; g.hitch@sussex.ac.uk

**Keywords:** travellers’ diarrhoea, acute diarrhoea, guidelines, pharmacy law, education, training, community pharmacy

## Abstract

International travel is growing and pharmacists are well placed to provide travel health services for the prevention and management of travellers’ diarrhoea (TD). Legislation changes in many countries has enabled pharmacists to access prescription only medicines and vaccinations to provide advice and over the counter medicines for the prevention and management for travel health services; this makes sense since pharmacies are easily accessible to the public and are the patient’s first port of call in the event of any illness. Currently, whilst many guidelines/guidance exist worldwide for the prevention and management of TD, there is no review that focuses on similarities and differences between these and between guidelines on TD and travel related and non-travel related acute diarrhoea. There is also a lack of publication on legislation and the need for evidence based training for all prescribers to provide travel health services. The aims of this work were to review guidelines/guidance for the prevention and management of TD from across the world which were compared with each other as were the TD guidelines compared to that for travel related and non-travel related acute diarrhoea for similarities and differences, with a focus on any relevant pharmacy legislation, needs assessments and training that may impact upon provision of travel health services by pharmacists focusing mainly on TD in adults. The PubMed, Google Scholar and Cochrane database were used to carry out an online search for publications on TD, acute diarrhoea and the guidance pharmacists have in the prevention and management of diarrhoea. The literature reviewed in this article indicates that where no specific guidelines/guidance existed, some pharmacists used the WHO guidelines (WHO), highlighting a need for local, regional and national evidence based guidelines in these countries.

## 1. Introduction

International travel has increased due to cheaper air fares making some of most seemingly inaccessible countries in the world to be reached relatively easily. In 2016, there were estimated to have been 58.2 million international tourists to Africa, 53.6 million to The Middle East, 302.9 million to Asia and Pacific, 200.9 million to the Americas and 619.7 million to Europe. Around 820.6 million tourists in total were estimated to have visited Europe and the Americas, and 414.7 million estimated tourists visited Africa and the Middle East [1]. According to the WorldAtlas website [2], the United States, China, Mexico and Thailand are the 3rd, 4th, 6th and 10^th^ respectively, most frequently visited countries by tourists all over the world outside of Europe. A survey by Geo-Sentinel data from 2007–2011 [3] from its 53 clinical sites in 24 countries (North America, Europe, Australasia, Latin America, Southern Africa and the Middle East) showed that the most frequent health hazards encountered by travellers included gastrointestinal and febrile systemic illness, and dermatologic diseases. Respiratory and ear–nose–throat was also identified amongst common infections acquired in a surveillance analysis undertaken in returning travellers in France from 2003 to 2015 [4]. In a study by Hill on factors that may affect the onset of TD in 784 American travellers who were provided with pre-travel advice, 46% reported having some form of diarrhoea of which 34% had TD [5]. TD is usually an acute, normally self-limiting condition which is rarely life threatening; however, it does affect the enjoyment of a holiday. According to the most recent guidelines by Riddle et al. [6] the stages of TD are defined as follows:

1. “Mild (acute): Diarrhoea that is tolerable, is not distressing, and does not interfere with planned activities.
2. Moderate (acute): Diarrhoea that is distressing or interferes with planned activities.
3. Severe (acute): Diarrhoea that is incapacitating or completely prevents planned activities; all dysentery (passage of grossly bloody stools) is considered severe; persistent: close space diarrhoea lasting two weeks”.

Table 1 shows the current guidelines/guidance that exist globally for the prevention and management of TD and acute diarrhoea [6,7,8,9,10,11,12,13,14,15,16,17,18,19,20,21,22,23,24,25,26,27,28].

Causes of TD can be bacterial, viral or parasitic. Bacterial TD is caused by *Escherichia coli* including strains of enterotoxigenic (ETEC) and enteroaggregative (EAEC) *E. coli*. [3,4,29,30,31,32] *Campylobacter, Salmonella* and *Shigella*, norovirus, astrovirus, *Giardia* and *Cryptosporidium* can all also cause TD with the former three microorganisms being the usual causes for TD along with EAEC [3,4,29,30,31,32]. Less common causes of TD include non-cholerae vibrios, *Aeromonas* species, and *Plesiomonas* species [8]. Travellers visiting Latin America also can have norovirus induced TD. Other TD causing viruses include astrovirus, rotavirus and adenovirus. Common parasitic infections that cause TD in travellers to Latin America and SouthEast Asia may be caused by *Giardia*, *Cryptosporidium* *and Entamoeba species* [3,8]. Chronic diarrhoea is usually caused by parasites in travellers on long term visits to developing countries. *Cryptosporidium parvum* with reported cryptosporidiosis TD has been reported in travellers to Russia [33]. A less common cause of TD is *Cyclospora cayetanensis* in travellers from Peru and Nepal [34].

Risk factors include destination, dietary habits, quality of local sanitation, age with younger adults and children who tend to be at higher risk [35] and the traveller’s susceptibility to infection [36] as well as patient factors such as altered upper gastrointestinal anatomy and the use of proton pump inhibitors or H_2_ receptor antagonists [9]. It was also interesting to observe that a study carried out on gender differences in travel-associated disease on data from the GeoSentinel network’s 44 sites which included 58,908 travellers, of which 50.3% were female and 49.7% were male, showed that female travellers were more likely to suffer from acute and chronic diarrhoea compared to men [37].

The risk and the causative organism of TD is highly associated with the travel destination as follows:

(a) low risk (North America, Western Europe, Japan, Australia and New Zealand);
(b) intermediate risk destinations include Russia, China, Eastern Europe, The Caribbean and South Africa;
(c) high risk destinations which include South and SouthEast Asia, Central America, most African countries, South America, Eastern Europe, and some Caribbean islands [10]. South and SouthEast Asia, Central America and West and North Africa are regions with particularly high risk of TD [8,10].

Haemolytic uraemic syndrome is a complication of TD caused by shiga toxin. Other complications of TD include *Campylobacter* induced Guillain Barré syndrome and in severe cases of TD, there is the potential for post-infectious arthropathies [38,39,40,41,42,43]. Protozoal infections caused by *Giardia* can also result in associated weight loss [43]. Another complication of post TD is irritable bowel syndrome (IBS) where studies have shown that up to 30% of the travellers experience IBS following TD [42,43].

## 2. Literature Review

**Figure 1:**
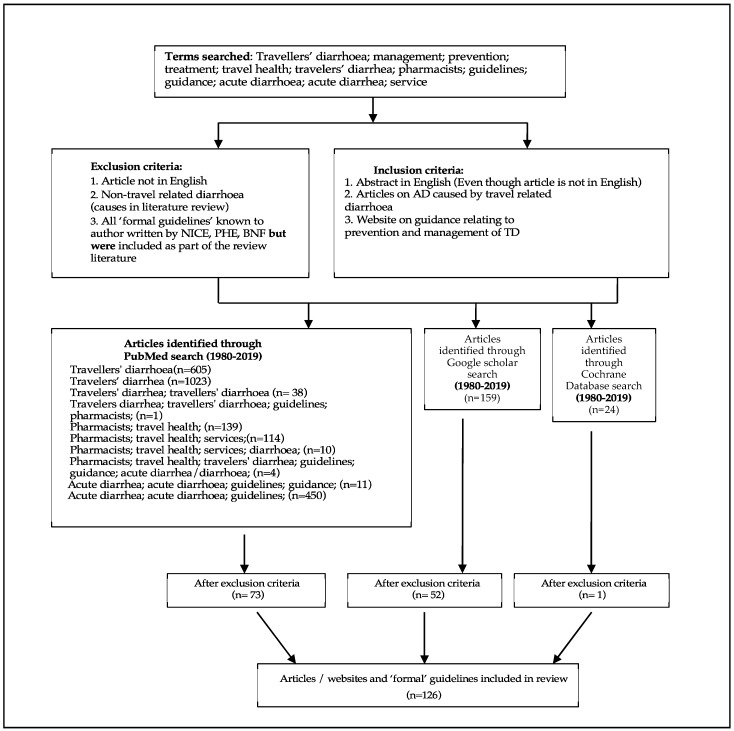
Flowchart showing search methodology.

The search terms used were travellers’ diarrhoea (UK and USA spelling); guidelines; pharmacists; acute diarrhoea (UK and USA spelling); treatment; prevention; management; travel health; services; (Figure 1 on Flow chart). The PubMed, Google Scholar and Cochrane database were searched for articles relating to the terms above from 1980 to 2019. Any article which did not include the term TD was excluded from the search as were the formal guidelines from the National Institute of Health and Care Excellence, Public Health England, The British National Formulary and the Health Protection Agency which the author was already aware of and included in this review. Any article which included the term ‘acute diarrhoea’ in the context of TD, only, was included, and this meant articles on diarrhoea caused by conditions such as ulcerative colitis, Crohn’s disease, irritable bowel syndrome (IBS), lactose intolerance, diabetes and pancreatitis were excluded. Articles relating to age-specific disorder causing chronic diarrhoea in children, or malnourishment related diarrhoea, mental health issues such as stress or anxiety and side effects of medicines such as cancer chemotherapy related causes of diarrhoea were all excluded from this review. Additionally, excluded from this search was any article not published in English but if a corresponding abstract was available in English this was included.

## 3. Review and Comparison of Guidelines/Guidance on Prevention and Management of TD Worldwide

Table 1 shows the guidelines/guidance for the prevention and management of TD. The most recent guideline for the prevention and management of TD is set by The International Society of Travel Medicine (ISTM) [6].

Table 1 also shows the Australian [11,12] and Canadian guidelines [13] for the prevention and management of TD. The differences between various guidelines lie in recommendations on the use of prophylactic antibiotics. For example, The Public Health England Antibiotic Guidance for Primary Care states that stand-by antimicrobials are indicated only for patients at risk of developing severe illness on a visit to high risk areas with a rare need for prophylactic antibiotics [14]. The American College of Gastroenterology (ACG) and the ISTM evidence based guidelines state that empirical use of antibiotics should be limited in the event of TD being of bacterial origin and the use of antibiotics should be dependent upon travel region and signs and symptoms of diarrhoea [7]. The National Institute for Health and Clinical Excellence’s (NICE) Clinical Knowledge Summaries (*CKS*) guidelines argue that travellers are not able to distinguish between bloody or non-bloody (non-invasive) diarrhoea, and as such, do not recommend the use of rifaximin but advocate the use of a fluoroquinolone such as ciprofloxacin to treat invasive diarrhoea [15]. Pharmacists also need to be aware of changes in antibiotic resistance patterns locally and look for evidence-based information when referring to any local or national guidelines.

The Australian evidence-based guidelines prepared by the Royal Australian College of General Practitioners (RACGP) recommend using oral rehydration salts to prevent dehydration followed by an antimotility agent, such as loperamide, which is preferred over the diphenoxylate/atropine (Lomotil^®^) combination due to its potential adverse effects profile [11]. These guidelines also recommend ciprofloxacin 500 mg or norfloxacin 400 mg as a single dose therapy or azithromycin 500 mg or 1000 mg as a single dose for the treatment of TD. An alternative treatment regimen includes a three day treatment of ciprofloxacin 500 mg or norfloxacin 400 mg every 12 h and azithromycin 500 mg once daily for three days in the event of infection with *Campylobacter* and *Shigella dysenteriae* where a single dose may not prove adequate. These guidelines also advise to stop taking any further antibiotics should TD resolve. It is interesting to note that this advice differs from the usual advice to complete a course of antibiotics and advises to keep the rest of the antibiotics for further treatment should TD reoccur. The RAGCP guidelines [11] do not advocate the use of routine antibiotic prophylaxis, but recommends the use of short courses of antibiotics for travellers at very high risk of developing infection and does not advocate the prophylactic use of rifaximin. Whilst this review has focused mainly on adults, it would be prudent for all healthcare professionals to be aware that the guidance for the use of antimotility agents differs for children in different countries. In the UK, children under 12 years [16] and in the USA children under two years [10] should not be treated with loperamide. Whilst the Australian guidelines indicate children under 12 years should not be given loperamide, many Australian doctors prescribe loperamide for children aged six years or older if required [11]. It is important to bear in mind that loperamide doses are different and vary according to the country’s licensing requirements. Pharmacists should consult latest online and/or reliable resources to verify the exact dose and frequency.

The Australian guidelines also recommend empirical self-treatment with tinidazole dose of 2 g (4 × 500 mg tablets) stat against *Giardia intestinalis* in travellers going on longer trips of 2–3 weeks. The directions for taking tinidazole are to take it after following a three day course of either fluoroquinolone or azithromycin antibiotic therapy, indicating a possible infection by the *Giardia* parasite if the duration of TD has been longer than 72 h. In the event of bloody diarrhoea and fever for 48 h, seeking medical guidance is recommended [12]. However, not all countries have specific guidelines on TD. Added to this, there is not much literature published on the management of TD or acute diarrhoea by pharmacists as stated previously in the literature review section.

## 4. A Review of Guidelines for the Prevention and Management Between Travellers’ Diarrhoea (TD) and Non-Travel Related and Travel Related Acute Diarrhoea

The authors who developed the guidelines for the prevention and management of TD have also developed the American College of Gastroenterology (ACG) clinical guideline on diagnosis, treatment, and prevention of non-travel related and travel related acute diarrheal infections in adults. Both guidelines bear some similarities in the management of travel related diarrhoea; however, they also indicate some therapeutic differences. The definition of TD [6] departs from the more traditional definition of TD as the authors feel that this system of definition is suitable towards a more tailored therapy for the prevention and management TD; whereas the definition for AD whether it is travel related or non-travel related describes it as a ‘passage of ≥3 unformed stools in 24 h plus an enteric symptom (nausea, vomiting, abdominal pain/cramps, tenesmus, fecal urgency, moderate to severe flatulence)” [7] p. 603. For the management of mild watery AD the ACG [7] recommends hydration through fluid and salt intake for all types of diarrhoea; the guidelines state that loperamide dose of 4 mg may be used initially to control stooling (maximum dose of 8 mg over 24 h period) whereas the ISTM [6] recommends use of rehydration with use of loperamide for the treatment of mild TD of 4 mg po then 2 mg after each loose stool not to exceed 16 mg daily alongside with hydration. (This dose and frequency is also similar to that recommended in other guidelines [12,16]. The ACG [7] also recommends loperamide (but not to be taken for more than 48 h) for the management of moderate to severe watery non-travel related diarrhoea, with low or no grade fever of <100°F (37.78 °C) diarrhoea. For moderate to severe watery non-travel related diarrhoea where fever is present for <3 days duration at >101°F (38.3 °C), loperamide is recommended but not to be taken for more than 48 h [7]. The ACG [7] also recommends that in moderate to severe watery non-travel related diarrhoea where fever is present for >3 days duration at >101°F (38.3 °C), microbiological assessment, and empirical therapy of azithromycin should be to be taken as 1 g in a single dose OR 500 mg once daily for three days. For the management of moderate to severe watery TD: ACG guidelines recommend a course of antibiotics. The ISTM guidelines recommend the use of loperamide as a monotherapy for moderate TD [6]. Both guidelines [6,7] recommend the use of bismuth subsalicylate (BSS) with the ACG guidelines stating the following dose for BSS in the treatment of mild to moderate AD: 30 mL (525 mg) of liquid formulation or two tablets (263 mg per tablet) chewed each 30–60 min maximum of eight doses in 24 h for prophylaxis, the ACG states that BSS can be given for a maximum of up to three weeks with a dose of two tablets four times a day at mealtimes and at bedtime for TD prevention according to ref by DuPont et al. [44,45]. ACG guidelines do not recommend empiric antimicrobial therapy for non-travel related AD and discourages the use of antibiotics in community acquired diarrhoea. Likewise, the ISTM guidelines do not recommend the routine use of antimicrobial prophylaxis and advocates antimicrobial prophylaxis only in high risk of groups at health-related complications of TD; rifaximin is recommended as a prophylactic agent and fluoroquinolones are not recommended for prophylaxis due to concerns over antibiotic resistance and associated adverse effects. ISTM [6] states caution against the use of fluoroquinolones in regions with high quinolone resistance and in SouthEast Asia, India or regions where fluoroquinolone-resistant *Campylobacter* or *resistant ETEC* has been identified; it also advises against the use of rifaximin in the event of invasive TD or in regions where this may be anticipated. Both guidelines recommend the use of antibiotics combined with loperamide for the management of moderate to severe diarrhoea. Whilst the ACG recommends a balanced ORS and medical evaluation in TD for the elderly in severe TD, the ISTM guidelines recommend the use of azithromycin to treat severe TD; fluoroquinolones to treat severe, non-dysenteric travelers’ diarrhoea and rifaximin to treat severe, non-dysenteric TD for the managment of dysenteric diarrhoea, both guidelines recommend the use of azithromycin 1 g in a single dose OR 500 mg once daily for three days [6,7]. Additionally, both guidelines advocate in cases of persistent diarrhoea (14–30 days), culture and/or culture-independent microbiologic assessment followed by treatment with antimicrobial agent directed to causal microorganism.

## 5. Antibiotics, Vaccines, and OTC Medicines for Prevention and Management of TD

### Antibiotics and Vaccines

Antimicrobial resistance is a worldwide problem. A recent study found that prescribing of stand by antibiotics led to travellers taking these even when TD was not severe [46]. This has the potential for travellers to become colonized by multidrug-resistant (MDR) intestinal extended-spectrum beta-lactamase-producing Enterobacteriaceae (ESBL-PE) [47] as well as shedding these bacteria on their return, and encouraging the spread of MDR bacteria in the community [47,48,49,50]. Enterotoxigenic *E. coli* that produce a heat labile (LT) or heat stable (STa) toxin are the most common species of *E. coli* implicated and the most prevalent cause of TD [51]. Either one or both strains of toxins have been isolated from patients with TD, depending upon which country they have visited [52]. Contaminated food and beverages are common sources of ETEC associated TD and currently the oral cholera vaccine, Dukoral^®^ [53] for protecting travellers against ETEC diarrhoea is available in the UK and Canada [13] for travellers if required. Although in the UK, whilst Dukoral is indicated for active immunisation in adults and children from two years of age travelling to or in cholera endemic areas against Vibrio cholerae serogroup O1, the indication does not specifically state it has a routine use in prevention of TD [53]. Research has shown that more data is needed to show its protective role in TD prophylaxis [54]. Similarly, a Cochrane review showed similar results of insufficient evidence from RCTs on the prophylaxis use of Dukoral for ETEC diarhhoea [51]. In the US, for adults aged 18–64 years who are travelling to an area of active toxigenic *Vibrio cholerae* O1 transmission the Vaxchora™ [55] single-dose oral cholera vaccine is recommended. This vaccine is approved by the US Food and Drug Administration.

Other MDR bacteria include Enterotoxigenic *Bacteroides fragilis* (ETBF) [56,57,58,59] and *Campylobacter jejuni* [60,61,62,63] are both enteric pathogen associated with TD. The reader is directed to a review by Hitch and Fleming [64] for an overview on antimicrobial resistance in TD.

The guidelines discussed in this paper, and listed in Table 1, recommend the use of azithromycin for the treatment of dysentery or bloody diarrhoea or against other bacterial infections, for regions where fluoroquinolone resistance to *Campylobacter* has been documented. The Public Health England Antimicrobial Prescribing Guidance [14] and NICE’s CKS [15] guidelines recommend the use of azithromycin in countries such as South and SouthEast Asia and in regions where there is known resistance to fluoroquinolones for preventative measures, and either ciprofloxacin (in regions other than where resistance to the drug is noted) or azithromycin only for the treatment of severe TD. All the guidelines recommend the use of azithromycin, which has a documented evidence base in areas where resistance to fluoroquinolones is prevalent and there is a high risk of invasive pathogens. Azithromycin is considered safe for use in pregnant women and young children for the treatment of severe TD. If during pregnancy or breast-feeding, treatment is required for acute diarrhoea, azithromycin or the less effective clarithromycin are recommended and for the treatment of TD caused by giardiasis, or amoebiasis, and metronidazole is recommended in preference to tinidazole [9].

Despite standard TD treatment, travellers on longer trips of more than two weeks, would benefit from metronidazole or tinidazole for treating noninvasive TD suspected to be caused by *Giardia intestinalis* or *E. histolytica* infection [65]. It would be prudent to seek medical confirmation of the cause of the TD only to rule out diarrhoea of non-infectious aetiology.

The FitforTravel, Health Protection Scotland’s guidelines, recommend rifaximin as a prophylactic antibiotic for at risk group of patients and either azithromycin, ciprofloxacin or rifaximin as a stand by treatment for severe diarrhoea [20]. Rifaximin is a non-systemic structural analogue of rifampin which inhibits the synthesis of bacterial RNA by binding to the b subunit of bacterial DNA dependent RNA polymerase. NICE’s CKS does not advocate the use of rifaximin for the treatment of severe TD [15]. The rationale behind this is that their guidelines are evidence based on several references [8,9,13]. Dupont’s study [66] states that as a chemoprophylactic for TD for trips ≤14 d, whilst rifaximin is safe as it is not absorbed, it is only moderately effective and literature review and research articles have shown that the efficacy of rifaximin is uncertain on invasive forms of TD caused by *Campylobacter* or *Salmonella*. In addition, some studies have shown that as a chemotherapy for TD, rifaximin is ineffective against mucosal invasive pathogens (*Shigella, Salmonella* and *Campylobacter*) [67,68,69,70]. However, rifaximin is approved for the treatment of TD caused by noninvasive enteric pathogens or *E. coli* infections in many countries worldwide [71]. Rifaximin is also not recommended as a prophylactic agent by the Australian guidelines [11,12]. The ISTM guidelines also present a weak recommendation with a moderate level of evidence, on the use of rifaximin for the treatment and prevention of TD [6]. A fluoroquinolone, azithromycin or rifaximin should be used for all other regions, and azithromycin should also be prescribed in the case of bloody diarrhoea. Prescribing of fluoroquinolones is recommended by all the guidelines in Table 1 for prophylactic measures for TD, except FitforTravel [20] (recommends rifaximin), Public Health England Antimicrobial Prescribing Guidance [14] (recommends azithromycin) and ISTM guidelines [6] (recommends rifaximin and azithromycin). The Sanford Guide recommends azithromycin 1 g once with loperamide on the onset of the first loose stool. For those with HIV and CD4 <200, going on short trips, it recommends ciprofloxacin 500 mg once daily or rifaximin 200 mg taken orally twice daily [28]. Articles reviewed here also show that pharmacists tended to refer to the International Travel and Health from the World Health Organization (WHO) website for Travel and Health guidelines [72] for the management of acute diarrhoea. For example, a study carried out in Trinidad using a simulated case study presentation of a patient with acute diarrhoea in community pharmacy with 92 pharmacists, showed 70% (n = 64) of pharmacists recommended ORS as a first choice therapy in children [73]. Over 60% recommended antimotility agents as a first choice of therapy or with ORS for adults and over 59% recommended co-trimoxazole for adults. Whilst the majority of pharmacists would provide information on constitution, preparation, storage and treatment schedule on ORS, few provided information on the discontinuation of ORS. Only 23 pharmacists adhered to the WHO guidelines [72] although the majority were aware of the existence of these guidelines. Another study aimed to assess travel health practices among community pharmacists in Malaysia and the relation to the quality of travel health services offered showed that out of the 111 respondents from 143 pharmacists to whom questionnaires were sent, 46% answered the only question on TD: *“Ciprofloxacin can be used as self-treatment for travellers’ diarrhoea in Thailand”* correctly as false, (p. 329) [74]. This study explored other broader topics on travel health services and so does not give a true picture of knowledge that Malaysian pharmacists may have in the field of TD. Their most widely used resource for travel health information however, was the WHO guidelines. South African pharmacists also have access to a wide variety of resources and guidelines such as Travax and the Centers for Disease Control and Prevention (CDC) website and WHO guidelines if they are a member of South African Society of Travel Medicine [75,76,77,78]. In another study in the USA, pre-travel services provided by pharmacists were compared to that provided by primary care physicians not trained in travel health medicine initially used the CDC guidelines and where these were unclear, referred to the WHO guidelines and Travax Encompass as secondary sources [79].

## 6. Updated Guidance on Use of Fluoroquinolone and Quinolone Antibiotics

Fluoroquinolones and quinolones are broad-spectrum antibiotics used to treat various infections. Ciprofloxacin is widely recommended by many guidelines (Table 1 for guidelines) for regions where resistance to fluoroquinolones is not documented. However, in terms of side effects and adverse drug reactions, it should be borne in mind by the pharmacist on how and what to counsel the traveller on, if a fluoroquinolone is prescribed, especially if it is someone in a high-risk group such as immunocompromised, pregnant, children, elderly or ongoing chronic diseases such as diabetes where glucose homeostasis can be adversely affected especially in patients on oral hypoglycemic agents [80].

The European Medicines Agency (EMA) has updated its advice on the use of fluoroquinolones [81]. The EMA’s human medicines Committee for Medicinal Products for Human Use (CHMP), recently endorsed the review of EMA’s safety committee Pharmacovigilance Risk Assessment Committee (PRAC) on restricting the use of these medicines for preventing TD. The review covered medicines containing the following fluoroquinolone and quinolone antibiotics such as ciprofloxacin, levofloxacin, moxifloxacin, nalidixic acid, norfloxacin and ofloxacin amongst others.

The CHMP has advised special caution in the elderly, patients with renal disease and organ transplantation due to being at higher risk of tendon injury. They also advise against the combined use of fluoroquinolones with corticosteroids as this increases risk tendon injury. Additionally, the British National Formulary (BNF) states that some common side effects can include a decrease in appetite, arthralgia, constipation, diarrhoea, eye disorders, gastrointestinal discomfort and QT interval prolongation to name a few. Other adverse effects can include Achilles tendon damage or *Clostridium difficile* infection.

There is also an increased risk of aortic aneurysm and aortic dissection in the elderly or in those at-risk and ligament rupture peripheral neuropathy, rhabdomyolysis and self-endangering behaviour [82,83]. The incidence or frequency of these side effects is unknown. However, the BNF [31] also recommends discontinuation in the event of psychiatric, neurological, or hypersensitivity reactions as well as if a severe rash occurs. Fluoroquinolones tends to upregulate cell matrix metalloproteinases and this causes types I and III collagen fibrils to be reduced. These collagen types are major components of the majority of collagen in both Achilles tendons and the aorta. This maybe a possible way by which the ruptures may be occurring [84]. Collagen also serves as a critical component of the vitreous body of the eye and in maintaining retinal attachment, but whether fluoroquinolones mediate retinal detachment is controversial even though eye disorders are listed as a side effect in the BNF [16].

### OTC Medicines

Severe dehydration with loss of fluids and electrolytes are lost during TD. This may also be accompanied by vomiting and its vital to replace the lost fluids. Pharmacists should advise travellers to drink lots of clear fluids, fruit juice or thin soups to maintain water and electrolyte balance. For at risk groups, in the children and the elderly, Oral Rehydration Therapy or Solution (ORT or ORS) is recommended to prevent complications from dehydration. Pharmacists should advise travellers on how to make their own ORS should none be available, by mixing six teaspoons of sugar and half a teaspoon of salt to one litre of clean water or lightly salted rice water, the recipe for which can be obtained from the WHO position paper on ORS [85] to reduce mortality from cholera. In the UK, ORS is available from pharmacies without a prescription as Dioralyte™ and Dioralyte Relief™ which contains pre-cooked rice powder, sodium citrate, sodium chloride, potassium chloride and the rice element helps the watery stools to return to normal and is used for rehydration and restoring electrolytes in TD [86]. Pharmacists should also make travellers aware of some ORS preparations that may contain a source of phenylalanine (aspartame) which may be harmful to people phenylketonuria. Some preparations contain ethanol (alcohol) which may be harmful to people with a drink problem, in pregnancy, breast-feeding or to children, or in conditions such as liver disease or epilepsy [86]. Pharmacists should also advise fully on the preparation of ORS and emphasize where drinking water is not available, the water should be freshly boiled and cooled and the solution should be made up immediately before use.

Loperamide has an antisecretory effect in low doses, that reduces the volume of watery stool. At higher doses, there is an additional antimotility effect. Evidence base for using loperamide suggests that it can reduce the duration and severity of TD [87]. Loperamide may be used if no red flag symptoms are identified in uncomplicated cases of TD (Table 2). It takes up to 1–2 h to work and cannot be given to children under four years old [16]. As stated previously, the doses for loperamide vary according to the licensing requirements for different countries. Pharmacists should be aware of this and make the traveller aware of this too depending upon the region of travel. Loperamide combined with simethicone is also available as Imodium Plus Caplets^®^ and Imodium Plus Comfort Tablets^®^ in the UK for painful wind and bloating with diarrhoea. A maximum dose of eight tablets or capsules can be taken over a 24 h period according to the UK licensing requirements. There are products one can buy from a pharmacy to help with wind, such as charcoal tablets or simethicone. A product that contains both loperamide and simethicone is available (Imodium Multi-Symptom Relief^®^) for added relief of wind. Loperamide can also be taken with antibiotics in the event of treatment of severe diarrhoea [88,89,90].

Other antimotility or antidiarrhoeal agents include diphenoxylate plus atropine (Lomotil^®^), and bismuth subsalicylate (BSS) (Kaopectate^®^ available in the USA, Pepto-Bismol^®^ available both in the UK and USA). BSS may cause darkening or blackening of the faeces and tongue along with constipation and possibly tinnitus. It should be avoided in people with allergy to aspirin, and in patients with renal insufficiency, gout, and by those on anticoagulants, probenecid, and methotrexate. BSS may enhance the effect of coumarin anticoagulants and oral hypoglycaemics of the sulphonylurea type. Salicylates also diminish the action of uricosurics [13,45,91]. Overdosing with aspirin or salicylates concomitant with the use of BSS may result in salicylate toxicity. BSS is not generally recommended for children aged <12 years in the USA and under 16 years in the UK [9,45,91].

The NICE-BNF guidance for children over three months and adults in the UK has listed racecadotril, which is an antisecretory drug licensed for the treatment of acute diarrhoea [92]. As no rigorous evidence-based clinical trials have emerged for the use of this drug in TD, it has been excluded from most guidelines on the prevention and management of TD. It is available on prescription only in the UK, and has been licensed for use in Europe, mainly in France for over 20 years and is recommended in the Consensus paper for the treatment of acute traveler’s diarrhoea and for the management of acute uncomplicated TD in children [19].

## 7. Aims of a Pre-Travel Consultation

The World Tourism Organization (UNWTO) is a specialized agency of the United Nations and data for 2018 showed that the number of tourists worldwide had risen by 6% in 2018 to 1.4 billion [93]. Using this data and given that the incidence for TD varies by destination, ranging between 10% and 40% [93], for travellers going to risk regions, between 140 million to 560 million cases of TD are likely to have occurred in 2018. Hence, it is vital for travellers to seek some form of travel health advice before travelling. The aims of the pre-travel consultation as stated by Hatz and Chen [94] should be based upon (1) the client’s fitness assessment for travel, and the type of travel (2) anticipated and real health risks and (3) subsequent prophylaxis measures to be taken. Historically, doctors and nurses have provided pre-travel consultation services as it was only these professions that were allowed to administer vaccinations [95]. However, over the last two decades, pharmacists have begun to provide these services as observed in many published articles on travel health services [96,97,98,99,100,101,102,103,104].

Many pharmacists also have access to guidelines as listed in Table 1 from various sources on the management of TD and acute diarrhoea. Whilst the aims of the treatment for AD is to prevent or reversal fluid and electrolyte depletion with the management of dehydration [16], in any patient group, the treatment should be tailored according to the severity of the symptoms.

## 8. Pharmacists’ Role in Prevention and Management of TD

Pharmacists are well placed and trained to provide advice, counselling and information on the side effects on taking antibiotics for the prophylaxis and treatment of TD. One of the most basic but extremely vital factors to consider is the extent in which travel risk assessment is carried out by the pharmacist, the current knowledge on the over the counter (OTC) medicines available for prevention and management of TD, as well as updates on guidelines as to where the traveller may be going. In terms of drug interactions, pharmacists should advise travellers if taking loperamide with or without simeticone, high doses of loperamide may cause serious cardiac adverse reactions such as QT prolongation, torsades de pointes, and cardiac arrest [12]. Pharmacists should be aware of the above drug interactions and the potential for constipation if used in conjunction with anticholinergics antispasmodics potent narcotic pain medicines, antihistamines, tricyclic antidepressants, cholestyramine, and antivirals [16].

Pharmacists should be vigilant about all OTC or POM products offered to the traveller since some may contain antacids or digestive medications and some combination products may also contain simethicone. Simethicone can also decrease the absorption of thyroid medications e.g., levothyroxine and pharmacists should advise travellers to leave at least 4 h between taking products that contain simethicone when taking thyroid medication [16].

Pharmacists should be aware and educate the traveller on any current medications they may be taking which can cause diarrhoea may also be a side effect of that medicine. For example, some antibiotics, any antacids that contain magnesium, some chemotherapy medicines, and non-steroidal anti-inflammatory drugs, statins and laxatives can all cause diarrhoea [16].

The main goal is to prevent dehydration via the adequate rehydration and supply of appropriate OTC medicines for symptomatic relief. Therefore, it is important for the pharmacist to be aware of signs and symptoms of TD and a good knowledge of what OTC products are available and how to use these along with raising awareness and providing appropriate counselling for the prevention and management of TD. In the case of the immunocompromised, pregnant, elderly or children who are regarded as ‘at risk’, the pharmacist should provide information ‘tailored’ to the traveller’s needs. If antibiotics are prescribed, counselling should be provided on potential drug interactions, taking adequate amounts of medication in order not to run out at the destination, educate the traveller on signs and symptoms of invasive diarrhoea and when to refer for medical care. Pharmacists should also educate the traveller on the awareness of ‘Red Flag’ symptoms in the event of being affected by TD (Table 2).

## 9. Advice on Best Practice

Pharmacists should educate travellers on using soap and water to cleanse hands after using the toilet and before and after preparing and eating meals. If there is lack of soap and water, then evidence has shown that alcohol-based hand sanitizers are also effective to reduce contamination on the hands. Pharmacists should advise on the use of hand rubbing gel sanitizers under the circumstances [105,106]. Advice on food and beverage consumption, storage, preparation and foods to avoid should also be provided. Whilst purified water is considered safe to drink, ice cubes made from local water is not safe to have in drinks. Ice cream and dairy products are also best avoided if there is no information on whether the milk has been pasteurised. Any fruits, vegetables or salads which are unclean or difficult to clean should also be avoided. Regarding the use of prebiotics and probiotics in the prevention or treatment of TD, lack of robust evidence has prevented these from being included in any of the mentioned guidelines [6,7,8,9,10,11,12,13,14,15,16,17,18,19,20,21,22,23,24,25,26,27,28]. Advice on best practice is best delivered if pharmacists have the proper communication and consultation skills in order to extract the history of travel, current medical conditions and medications, allergies, and appropriate qualification for prescribing, and clinical training in prescribing, supplying and administration of medicines and vaccinations. During the pre-travel assessment, pharmacists should ask probing questions around the destination, duration of travel, standards of hygiene and sanitation at the place of stay. They should also explore how travellers intend to take practice and take precautions against local food, water, and personal hygiene, traveller’s own risk and assessment of TD and risk of complications, access to local medical facility and planned journeys and necessity of travel to high risk areas. There are many publications and guidelines that highlight the possibilities of acquiring TD whilst travelling from developed countries to developing countries, but a recent study on travellers who travelled to developed countries in North America, Australia, Europe and New Zealand, found that 24 out of 99 travellers (24%) had gastrointestinal related diarrhoea conditions of which 62% were Australian residents, 24% were residents of other ‘developed’ countries, and the remaining 14% were residents of other countries [107]. Travellers generally do not seek advice prior to visiting developed countries. It is therefore important that when travellers come to the pharmacist adequate advice regarding the destination or region of travel should be provided.

## 10. In Summary

In summary, the key findings of this review include the following:

1. Guidelines/guidance were found online for the many developed countries.
2. There are some differences in guidelines/guidance between the prevention and management of TD and acute diarrhoea with a single difference of a maximum dose of loperamide of 8 mg for acute diarrhoea and 16 mg for TD.

## 11. Conclusions

Comparison between the guidelines from various countries on the prevention and management of TD showed many similarities regarding recommendations on the use of quinolones/fluoroquinolones in regions where resistance was /is reportedly high to this class of drugs. There was some variation on the use of antibiotics as a prophylactic and as stand by medicines. There was some difference in the guidelines between the prevention and management between TD and acute diarrhoea in the case of maximum loperamide dose over a 24 h period. There is a dearth of literature specifically directed towards the role of pharmacists in the prevention and management of TD.

## Figures and Tables

**Table 1:** Guidelines or guidance from various countries for the prevention and management of TD.

Guidelines	Country
Riddle, M.S.; Connor, B.A.; Beeching, N.J. et al. Guidelines for the prevention and treatment of travelers’ diarrhea: A graded expert panel report. *J. Travel Med.* 2017; 24. S63–S80 [6].	USA
Riddle, M.S.; DuPont, H.L.; Connor, B.A. ACG clinical guideline: diagnosis, treatment, and prevention of acute diarrheal infections in adults. *Am. J. Gastroenterol.* 2016. 111. 602–22 [7].	USA
Barrett, J. and Brown, M. Travellers’ diarrhoea. BMJ. 2016. 353. 1937. doi:10.1136/bmj.i1937 [8].	UK
Barrett, J. and Brown, M. Diarrhoea in travellers. 2018. Medicine 46. 1. 24–29 [9].	UK
Bradley, A.C. Travelers’ Diarrhea: Centers for Disease Control and Prevention; 2017. Available from: wwwnc.cdc.gov/travel/yellowbook/2018/the-pre-travel-consultation/travelers- diarrhea (accessed 1 March 2019) [10]	USA
Leder, K. Advising travellers about management of travellers’ diarrhea; The Australian Family Physician. 2015 44. 1. 34-37. (Royal Australian College of General Practitioners (RACGP) [11] https://www.racgp.org.au/afp/2015/januaryfebruary/advising-travellers-about-management-of-travellers%E2%80%99-diarrhoea/ (accessed 1 March 2019)	Australia
Expert Group for Antibiotic. Antibiotic: Gastrointestinal tract infections: Acute gastroenteritis: Acute diarrhoea in special groups: Travellers’ diarrhoea. In: eTG Complete [Internet] Melbourne. Therapeutic Guidelines Ltd. 2014 [12]	Australia
Libman, M. Committee to Advise on Tropical Medicine and Travel (CATMAT). Summary of the Committee to Advise on Tropical Medicine and Travel (CATMAT) Statement on Travellers’ Diarrhea. Can Commun Dis Rep. 2015.41.11.272-85. [13] https://www.canada.ca/en/public-health/services/reports-publications/canada-communicable-disease-report-ccdr/monthly-issue/2015-41/ccdr-volume-41-11-november-5-2015-foodborne-illness/ccdr-volume-41-11-november-5-2015-foodborne-illness-2.html (accessed 1 February 2019)	Canada
National Institute of Health and Care Excellence, Public Health England. Summary of antimicrobial prescribing guidance – managing common infections. [14] https://www.gov.uk/government/publications/managing-common-infections-guidance-for-primary-care (accessed February 2019)	UK
The NICE CKS Diarrhoea - prevention and advice for travellers’ diarrhea—Prevention and advice for travellers. 2018. [15] Available from: https://cks.nice.org.uk/diarrhoea-prevention-and-advice-fortravellers#!topicsummary. (accessed 1 March 2019)	UK
British National Formulary. 2019. 76th Edition. London: British Medical Association and Royal Pharmaceutical Society. (accessed 1 March 2019) [16]	UK
Médecins Sans Frontières. Clinical guidelines - Diagnosis and treatment manual. 2018 edition. *ISBN 978-2-37585-027-5* https://medicalguidelines.msf.org/viewport/CG/english/acute-diarrhoea-16689593.html (accessed March 2019) [17]	France
Marchou B. Travellers’ diarrhea: Epidemiology, clinical practice guideline for the prevention and treatment. Presse Med. 2013. 42. 1. 76–81. doi:10.1016/j.lpm.2012.10.008 [18].	France
Jelinek, T.; Nothdurft, H.D.; Haditsch, M. et al. Consensus paper treatment of acute traveler’s diarrhea. Practice recommendation for travel advice. MMW Fortschr Med. 2017. 159. 4. 4-11. [19].	Germany
Health Protection Scotland, NHS National Services Scotland. [20]. http://www.fitfortravel.nhs.uk/advice/disease-prevention-advice/travellersdiarrhoea. (accessed March 2019)	Scotland
Gastroenteritis (Food Poisoning). 2018. Department of Family Medicine, Singapore General Hospital. [21] https://www.singhealth.com.sg/patient-care/conditions-treatments/gastroenteritis-food-poisoning (accessed 1 March 2019)	Singapore
‘I have travelers diarrhea, what should I do? Should I see a doctor? Thai Travel Clinic, Hospital for Tropical Diseases, Faculty of Tropical Medicine, Mahidol University. [22] https://www.thaitravelclinic.com/blog/health-problem/i-have-travelers-diarrhea-what-should-i-do-should-i-see-a-doctor.html (accessed 1 March 2019)	Thailand
Health Protection Agency. Foreign travel-associated illness – a focus on travellers’ diarrhoea. 2010 report. London: Health Protection Agency; 2010. (accessed 1 February 2019) [23]	UK
GPN update: Travellers’ diarrhoea: Prevention and management. [24] https://www.gpupdate.co.uk/SM4/Mutable/Uploads/pdf_file/Travellers_ diarrhoea_August2016.pdf (accessed 1 March 2019)	UK
NaTHNac guidance on Travellers’ diarrhoea. [25] https://travelhealthpro.org.uk/factsheet/53/travellers-diarrhoea (accessed 1 March 2019)	UK
National Institute of Health and Care Excellence. Treatment summary for acute diarrhoea. [26] Available from: https://bnf.nice.org.uk/treatment-summary/diarrhoea-acute.html (accessed 1 March 2019)	UK
The Centers for Disease Control and Prevention (CDC) yellow book. [27] Available from: https://wwwnc.cdc.gov/travel/yellowbook/2018/the-pre-travel-consultation/travelers-diarrhea (accessed 23 April 2019)	USA
Gilbert, D. N., Chambers, H. F., Eliopoulos, G. M., Saag, M. S., & Pavia, A. (2019). *Sanford guide to antimicrobial therapy 2018.* 49th edition; mobile app edition. Sperryville, VA, USA: Antimicrobial Therapy, Inc. [28]. Obtained as a mobile App from AppStore. Updated 9th April 2019. (accessed 23 April 2019)	USA

**Table 2:** ‘Red flag’ symptoms for doctor’s referral.

Medical co-morbidities, particularly immunosuppression or gastrointestinal disorders
Blood in stools: Dark or black stools—This may be a sign of bleeding inside the stomach
Severe or continuous abdominal pain
High fevers
Children and older people
Weight loss
Persistent vomiting
Signs of dehydration: Drowsiness; Passing urine infrequently; Feeling lightheaded or dizzy; Feeling thirsty; A dry mouth; Lethargy; Having dark coloured, strong-smelling urine
